# Supplementary material for: The genome sequence of the Carline Skipper, Pyrgus carlinae (Rambur, 1839) (Lepidoptera: Hesperiidae)
Source: Wellcome Open Res. 2026 Jan 20;11:50. [Version 1] doi: 10.12688/wellcomeopenres.25810.1 (PMC12989058; doi:10.12688/wellcomeopenres.25810.1)
Supplement: Peer review history

# Reviewer report: Reviewer response for version 1

**DOI:** 10.21956/wellcomeopenres.28411.r149479

**Published:** 2026-03-14

**Authors:** Laura Jimeneza Burney

**Affiliations:** 1 University of Liverpool, Liverpool, UK

This report describes the high-quality-level genome assembly for a female *Pyrgus carlinae* (Carline Skipper), a Lepidoptera species endemic to the western and south-western Alps. The primary assembly (Haplotype 1) spans 808.07 Mb and has been scaffolded into 25 chromosomal pseudomolecules, which include both the W and Z sex chromosomes. This work, conducted as part of Project Psyche, utilized PacBio HiFi long-read sequencing and Hi-C chromatin conformation capture to achieve high continuity and accuracy.

- The authors report a substantial difference in span (808.07 Mb vs 554.08 Mb) and BUSCO completeness (98.4% vs 74.9%) between Haplotype 1 and Haplotype 2. While the focus is primarily on the chromosome-level Haplotype 1, the authors do not provide a technical explanation for why Haplotype 2 is significantly less complete. This information is important for understanding the assembly outcome and should be included.
- In the *Methods* section, the authors provide detailed collection data and a voucher photograph (Figure 1). However, they do not state where the physical specimen has been deposited. For the sake of long-term verification, the authors should explicitly state the public collection or museum where the voucher specimen (or its remains) is housed.
- In the *Hi-C library preparation and sequencing* section, the authors write: "create equimolar and/or weighted 2.8 nM pools". The term "weighted" is unclear. If the authors mean that the pooling was adjusted based on specific criteria such as estimated genome size or library yield, this should be stated explicitly rather than implied through the pooling description.
- With regards to genome annotation, the authors state that the average transcript length is 20,934.66 bp. If "transcript" refers to processed mRNA, this length is unusually large and more consistent with the total gene span including introns. The authors should clarify whether 20,934 bp refers to the mRNA transcript length or the gene length.
- The authors state that **MitoHiFi** was used "to ensure the general quality of the sequence". "General quality" is a vague description of the results. The authors should provide specific metrics, such as circularization status or a completeness comparison against a reference mitogenome, to substantiate the quality of the mitochondrial assembly.

Are sufficient details of methods and materials provided to allow replication by others?

Yes

Is the rationale for creating the dataset(s) clearly described?

Yes

Are the datasets clearly presented in a useable and accessible format?

Yes

Are the protocols appropriate and is the work technically sound?

Yes

Reviewer Expertise:

Population genetics, evolutionary biology, conservation genomics

I confirm that I have read this submission and believe that I have an appropriate level of expertise to confirm that it is of an acceptable scientific standard.

# Reviewer report: Reviewer response for version 1

**DOI:** 10.21956/wellcomeopenres.28411.r145598

**Published:** 2026-02-10

**Authors:** Ian M Carr

**Affiliations:** 1 University of Leeds, Leeds, UK

**The genome sequence of the Carline Skipper, *Pyrgus carlinae* (Rambur, 1839) (Lepidoptera: Hesperiidae)**

- Although genome assembly is a computationally intensive process, the authors do not describe the operating system used or the computational resources (RAM, CPU cores, or runtime) required for key steps such as the assembly. This information is important for reproducibility and should be included.
- In the *PacBio HiFi library preparation and sequencing* section, the authors state: “The kit includes reagents for end repair/A‑tailing, adapter ligation, post‑ligation SMRTbell bead clean‑up, and nuclease treatment.” This is a description of the kit, not of the procedures performed. The authors should explicitly state that they carried out end repair, A‑tailing, adapter ligation, etc., rather than implying it through the kit contents.
- When the Femto Pulse system is first mentioned, the manufacturer and model are not provided, but they are included on the second mention. These should be swapped so that the full details appear at first mention.
- In the *Assembly curation* section, the authors write: “The assembly was decontaminated.” The term *decontaminated* typically refers to removal of hazardous substances. A clearer phrasing would be: “Extraneous or contaminant sequences were removed.”
- The authors state: “Sequences without hits are chunked.” The term *chunked* is unclear. If they mean that sequences were split into smaller batches (e.g., using **seqtk**) prior to BLAST searches, this should be stated explicitly.
- In the first paragraph of the *Genome assembly* section, the authors describe using k‑mer analysis to estimate genome size, but they do not reference Figure 2, which displays the k‑mer distribution. Figure 2 should be cited here.
- In the final paragraph of the *Assembly quality assessment* section, the authors describe the use of **lep_busco_painter**, but do not mention that Figure 4 shows the resulting plot. Figure 4 should be referenced in this paragraph.
- In Figure 6, the BUSCO categories are displayed as a circular plot from 0–100%, but the values in the key sum to 102.1%. The “Comp (98.4%)” value appears to include single‑copy, duplicated, and fragmented genes, even though the latter two categories are also shown separately. “Comp” should instead reflect only the single‑copy BUSCOs (96.35%). The abbreviations “Comp”, “Frag”, “dupl”, and “missing” should also be defined. Additionally, the tick marks in the lower‑left scale are rendered at a single‑pixel width due to monitor antialiasing, making them visually indistinguishable; consider enlarging them, referencing larger segments, or removing them.
- In the *Genome annotation report* section, the authors state that the average transcript length is 20,934.66 bp. If “transcript” refers to processed mRNA, this length is unusually large and more consistent with gene length or unprocessed pre‑mRNA. The authors should clarify whether 20,934 bp refers to the mRNA transcript length or the gene length. They also state that the average gene has 1.6 transcripts; it would be clearer to say that each gene has an average of 1.6 splice variants or transcript isoforms.

Are sufficient details of methods and materials provided to allow replication by others?

Yes

Is the rationale for creating the dataset(s) clearly described?

Yes

Are the datasets clearly presented in a useable and accessible format?

Yes

Are the protocols appropriate and is the work technically sound?

Yes

Reviewer Expertise:

Bioinformatics

I confirm that I have read this submission and believe that I have an appropriate level of expertise to confirm that it is of an acceptable scientific standard.
